# Rare disease 101: an online resource teaching on over 7000 rare diseases in one short course

**DOI:** 10.1186/s13023-024-03286-8

**Published:** 2024-07-22

**Authors:** Thomas Frederick Dunne, Daniel Jeffries, Lucy Mckay

**Affiliations:** 1 Alder Hey Children’s NHS Foundation Trust, Eaton Road, Liverpool, L12 2AP UK; 2 Medics4RareDiseases, Unit 12 Treadaway Technical Centre, Treadaway Hill, Loudwater, High Wycombe, HP10 9RS UK

**Keywords:** Rare disease, Genomics, Patient experience, Education, Online education, Learning management system, Interactive learning, Advocacy, Patient expert

## Abstract

**Background:**

An estimated 3.5 million people in the UK live with a rare disease however due to the rarity of each individual condition this is not currently reflected in mainstream medical education. As a result, common features of living with a rare condition include diagnostic delay, poor coordination of health and social care and lack of access to specialist care and treatment. This is well documented in reports published by patient advocacy groups collating the patient experience and has been highlighted by the Department of Health and Social Care in its UK Rare Diseases Framework. One of the four priority areas outlined in this policy published in 2021 is ‘increasing awareness amongst healthcare professionals’.

Medics4RareDiseases (M4RD), a charity based in the UK, has proposed a disease-agnostic approach to educating doctors about rare disease, focusing on the common challenges experienced across this heterogeneous collection of conditions, rather than on the minutiae of each of the > 7000 rare conditions. A literature search using MEDLINE, PubMed Central and Bookshelf confirmed a lack of broad rare disease teaching in medical literature; none of the 10 final resources identified focused on the topic as a whole.

**Results:**

To address this, M4RD created the course ‘Rare Disease 101’. It is accessed online using a learning management system that is free, contains interactive lessons, hosts a discussion board and is easily updated. In the 29 months since going live, 942 individuals have registered with 204 having completed the course; early feedback from 33 respondents was unanimously positive (all participants rated at least good (76%: excellent)) demonstrating that both clinicians and patients can benefit from broad rare disease education. The course is freely available to all at https://learn.m4rd.org/.

**Conclusions:**

Disease-agnostic training about rare disease as a large patient population, focusing on its unique profile of unmet needs, is required. Rare Disease 101 provides a pragmatic approach to an educational challenge that leads to poor patient outcomes. Early results suggest that the educational programme is well-received but further evaluation and assessment is needed.

**Supplementary Information:**

The online version contains supplementary material available at 10.1186/s13023-024-03286-8.

## Background

### Rare disease medicine

A rare disease is any condition with a prevalence of less than 1 in 2000 [1, 2]. Over 7000 have been identified to date, with this number growing [2, 3]. Although individually rare, they are collectively common with global rare disease point prevalence estimated at 3.5–5.9% [4]; this equates to approximately 3.5 million people in the UK [5, 6]. Although a clinically heterogeneous population, this commonality refers to a shared patient experience that includes a lack of available information, diagnostic delay and limited treatment options [7, 8]. Rare diseases can be found in every area of medicine; they present across all ages and demographics however their individually low prevalence means they are not an educational priority. Clinicians cannot know all rare diseases, but with 3.5 million affected patients, they need a fundamental understanding of the field. Serial reports indicate that inadequate knowledge and misconceptions about the prevalence of rare diseases result in suboptimal patient care, compounded by insufficient formal medical education [5, 9, 10]. On average, it takes over four years for a patient to receive a diagnosis, seeing four primary care doctors and four specialists during that time [11, 12]. This delay causes significant distress to patients and their loved ones, and is costly to the National Health Service (NHS) [12]. Furthermore, these conditions often carry significant burden of disease and/or disability, with 30% of those with a rare disease dying before their fifth birthday [5, 13].

### Rare disease education and strategy

It has been recognised that education is lacking in the field of rare disease throughout undergraduate and postgraduate medical training [8, 14–16]. Where available, such education focuses on the details of individual conditions as a vehicle for teaching about specific principles such as genetic patterns of inheritance or metabolic pathways. This leaves clinicians with gaps in their knowledge and practical skills when managing the presentation of rare disease in clinical practice [17]. 

The gap in rare disease education is not new, but improving knowledge in this area is increasingly important due to significant advancements in diagnosing and treating rare conditions over the past two decades. Developments in genomics, such as the Diagnosing Developmental Delay study and the 100,000 Genomes Project, have pushed the rare disease field into the spotlight. Approximately 80% of rare diseases have a genetic component [15] and these improvements in diagnostic power have facilitated one in four UK patients who took part in the 100K Genome Project receiving a diagnosis [5]. With an increase in the rate of diagnosis, clinicians can improve management for more patients. However, more people with rare diseases also translates to increased pressure on the NHS, requiring novel pathways, systems and infrastructure. This has led to the development of guidelines and frameworks to improve rare disease education and resource availability for clinicians addressing these challenges [2, 5, 18–20]. 

January 2021 saw the publishing of ‘The UK Rare Diseases Framework’ by the UK Department of Health & Social Care [5], replacing the UK Rare Diseases Strategy from 2013 [8]. This framework has four priorities: 1. ensuring patients get the right diagnosis faster; 2. increasing awareness of rare diseases among healthcare professionals; 3. better coordination of care; 4. improving access to specialist care, treatment and drugs. These priorities are disease-agnostic, referring to the shared challenges of the rare disease population. The document explains the direct impact that clinician education will have on priority 2, whilst indirectly addressing priorities 1, 3 and 4. This highlights the importance of educating healthcare professionals in rare diseases to UK health goals. Through provision of education to clinicians on diagnosis and management of rare disease, the time to diagnosis (often termed the ‘diagnostic odyssey’) can hopefully be shortened. This in turn should lead to: 1. an improved patient journey; 2. better patient-doctor relationship; 3. an earlier start point for patients to plan care/treatment; 4. a reduction in unnecessary investigations; 5. reduced cost to the NHS.

### Rare disease education in literature

To understand what current rare disease education is available, a literature review of articles was conducted using the PubMed search engine of MEDLINE, PubMed Central and Bookshelf. Articles published up to June 2021 were searched utilising the strategy ‘(rare disease*) AND (education OR teach*)’, delivering 8557 English language results with full text available. These were explored manually, initially by title, then by abstract, which resulted in 10 publications that regarded an educational resource on a specific rare disease therapy. These educational resources however did not relate to the management of rare/undiagnosed diseases as a general topic but identified specific diseases/learning situations. The topics identified were: genetic testing [21], systemic sclerosis [22], Moebius syndrome [23], primary immunodeficiency [24], malignant hyperthermia [25], Fabry disease [26], Hunter disease [27], and rare disease prevalence [10]. The final two publications related to Ebola Virus Disease, a rare infectious disease [28, 29]. A search utilising Google Scholar/Google at the time of this publication identified resources available to educate on genomic and translational medicine through the Health Education England Genomics Education Programme [30] and the Genetic and Rare Diseases Information Centre [31], and access to online courses on rare disease medicine through the Undiagnosed Diseases Network International (UDNI), the European Joint Programme on Rare Diseases (EJP RD), the National Organization for Rare Disorders (NORD) and Rare Disease Europe (EURORDIS) [32–35]. The UDNI site has one active link to an educational course: Rare Disease 101 (the subject of this article) [32]. The EJP RD have published two out of a planned four online educational academic courses on aspects of rare disease research; each course runs over a five-week period with three-four hours of work per week [33]. NORD hosts three courses of which one is specific to rare disease drug development [34]. EURORDIS hosts a number of courses under the themes of ‘research’, ‘diagnosis’ and ‘holistic care’ in rare disease medicine [35]. These research-orientated courses range from one hour to nearly six hours completion time, and contain a mixture of videos, reading and short quizzes.

To further explore what educational resources are available to UK clinicians on the general approach to managing rare and undiagnosed diseases, searches were performed of commonly used online platforms, including Patient.info [36], BMJ Best Practice [37], Uptodate [38], GP notebook [39] and NICE CKS [40]. Patient.info, BMJ Best Practice and Uptodate provide details on specific rare conditions but lack guidance on the holistic management of patients with rare or undiagnosed diseases. Genetics and genomics were better represented with information on all five sites including specific conditions with genetic components [36–40], personalised medicine [38] and genetic counselling [39]. These searches highlight a limited availability of clinically-orientated educational resources on management of rare and undiagnosed diseases.

### Medics 4 rare diseases

Medics 4 Rare Diseases (M4RD) is a charity registered in England and Wales that provides education in the field of rare diseases for medical students and doctors in training. It strives to drive an attitude change towards rare disease amongst medical professionals to reduce the diagnostic odyssey and improve the patient experience. The charity has been hosting events with a rare disease focus since 2011. In 2014, they partnered with the Medical Genetics Section of The Royal Society of Medicine (RSM) to hold their first joint symposium, "The Unusual Suspects: Rare Disease in Everyday Medicine." This annual event aims to provide a basic understanding of important and generalisable concepts of rare disease medicine through a short afternoon of lectures. It has been popular and well-received, with a 2024 Net Promoter Score of 62. This concept was the precursor to development of the e-learning tool. On February 16th 2021 the charity published Rare Disease 101, an online course on the recognition and management of rare disease for clinicians and medical students. It is available online at https://learn.m4rd.org/. This tool uses a Learning Management System (LMS) in order to allow ease of distribution to a large audience, equity of access, flexibility of completion and ease of feedback collection and collation. It aims to address the gap in rare disease education for medical students and clinicians. This article will describe the tool's development, present its current form, and discuss early feedback.

### Aims

- To present and illustrate Rare Disease 101, an online course (hosted through an LMS) providing a series of lessons to educate clinicians on the management of rare and undiagnosed diseases.
- To review early feedback from users of the learning tool.

## Methods

### Development

Development of Rare Disease 101 stemmed from the joint symposium between M4RD and RSM, ‘The Unusual Suspects: Rare disease in everyday medicine’. From the first meeting, these events promoted a disease-agnostic approach to rare disease education. The speakers were not invited to talk about individual conditions as such, but to explain broader issues and experiences, for example the diagnostic odyssey. Every event included patients, advocates, clinicians, medical students and someone involved in research or innovation. The lived experience is very much at the heart of these meetings.

Rare Disease 101 was created by taking this formula and designing content that provided the same education in an engaging and accessible way when accessed via an LMS. Thought leaders with expertise in different areas of rare disease were invited to write the content of the lessons alongside a member of the M4RD team. These organisations/individuals are detailed in ‘Additional File 1’. Multimedia resources from all over the rare disease community were then incorporated into the lessons.

The eight lessons included in Rare Disease 101 were chosen based on the most common themes that emerged from ‘The Unusual Suspects’ over the years and influenced through consultation with other stakeholders in the rare disease field. The course in its entirety is expected to take around two hours to complete. Table 1 details the eight lessons, along with a summary of their content and examples of feedback. The feedback included is not exhaustive but illustrates examples that generated the lesson themes and informed the associated content.

**Table 1:** The eight lessons of rare disease 101

Lesson	Content	Reason
Introduction to Rare Disease	Definition of a rare disease Prevalence of rare disease Aetiology of rare disease Relevance of rare disease	“[I found helpful] Importance of rare diseases in clinical practice” RSM 2017, feedback “This is the first time I've attended an event on rare diseases and at first I really wasn't sure what to expect but having attended I have had a great insight into rare diseases and learnt a lot” RSM 2017, feedback “Relevance of rare disease to my everyday practice” RSM 2019, learning need “Understanding of how rare disease affects doctors from across the medical profession” RSM 2019, learning need “An awareness of the size of the RD problem” RSM 2020, learning need
The Diagnostic Odyssey	What is meant by the term ‘Diagnostic Odyssey’ Why a rare diagnosis might be delayed The impact of the diagnostic odyssey on patients, families and health systems The benefits of a timely diagnosis in rare disease Resources and approaches for reducing the diagnostic odyssey	“[I would like to know more about] how we need to work on the diagnostic challenges surrounding rare disease” RSM 2017, learning need “To consider a diagnosis of rare diseases in patients with symptoms that persist or don't match up” RSM 2017, take away “This conference will empower me to consider rare diseases in the future when making a diagnosis, and not shy away from them because of a lack of understanding or knowledge of the specific disease” RSM 2019, feedback “A better understanding of how to spot rare diseases, and what to do when you think a patient may have one but you don't have detailed/any knowledge about the specific disease in question. Dealing with this uncertainty in a productive way!” RSM 2019, learning need “More information about how rare diseases are diagnosed and how it affects the patients life” RSM 2019, learning need
Sarah’s story (part of lesson 2)	A first-hand account of a patient journey	“I was really moved by her story” RSM 2020, feedback “Have seen her speak about the book before and have read it, and her talk was still interesting” RSM 2020, feedback “Wonderful advocacy—bravo Sarah” RSM 2020, feedback
Understanding the Common Challenges	The global challenges associated with rare diseases Diagnostic and management challenges Psychosocial challenges Short- and long-term strategies for managing patients	It has opened up my mind to many conditions I didn't know about before and how these conditions can affect patients and their families, not just the disease itself but also everything else. I am now more inclined to find out more about rare diseases and hold a different communication approach in practice” RSM 2017, feedback "A better understanding of how to manage patients with rare diseases” RSM 2017, learning need “Better aware rare disease affects 1 in 17…importance of having someone who can co-ordinate care (GP) and manage general symptoms” RSM 2019, feedback “Better understanding of the role of patients in research” RSM 2020, learning need
Diagnostic Tools	Red flags for a possible undiagnosed rare disease Accessible diagnostic tools Key principles in searching for a diagnosis Emerging tools to assist diagnosis	hat are the key triggers to start to think outside the box” RSM 2017, learning need “An understanding of advances in the field” RSM 2019, learning need “Where we are with diagnostics with rare diseases in UK and new projects” RSM 2020, learning need
The Role of Genomics	Genetic testing, exome sequencing and whole genome sequencing 100K Genomes Project The NHS Genomics Medicine Service The Genomics Education Programme	“An insight into rare genetic diseases” RSM 2017, learning need “New knowledge of diagnosis rare diseases and role of 100K genome project” RSM 2019, feedback “Ideas and where to find information about rare diseases learn about nhs genomics programme” RSM 2019, learning need “Update on 100K genome. Further insight in rare diseases from patient and healthcare professional perspective. Need to introduce education on rare diseases at medical student level” RSM 2019, learning need “Better understanding of mainstreaming of genomics at the coal face” RSM 2019, learning need
Patient Advocacy Groups	What patient advocacy groups are and why they are needed in rare disease Services provided by patient groups to patients, families and healthcare professionals Case studies of patient groups (from established charities to kitchen table support groups)	“[I would recommend] the opportunity for students to hear from other patient groups” RSM 2017, feedback “Curious about what rare diseases organisations do and whether there is opportunity for myself to be involved” RSM 2017, learning need “Really enjoyed the presentation from patients and patient groups, and would like more of those” RSM 2017, learning need “Latest advancements in rare diseases, patient advocacy groups,…” RSM 2024, learning need
Supporting Patients for Better Outcomes	The role of good communication in coordinating patient care and supporting families Effective communication and listening techniques Lines of communication when dealing with patients and caregivers Reflection on current communication skills and areas for improvement	“[I found helpful] topics touched on around communication and consider how difficult it must be to not have a diagnosis due to delays in reaching this point” RSM 2017, feedback “I hope to learn more about how to deal with patients that may present with a rare disease” RSM 2017, learning need; “An appreciation of the best support for patients with rare diseases” RSM 2020, feedback A better understanding of the role of patients in research” RSM 2020, learning need
Education	Educational rare disease resources Opportunities for healthcare professionals in rare disease	“More information about rare disease” RSM 2017, learning need “Where we are with diagnostics with rare diseases in UK and new projects” RSM 2020, learning need “…how people can educate themselves on other rare diseases” RSM 2024, learning need

The eight lessons of Rare Disease 101 alongside (1) a short summary of the content of each lesson, (2) examples of feedback and attendee-identified learning needs that directed content development

The course was created using H5P hosted on WordPress by a Trustee of M4RD who himself has two rare conditions, Dan Jeffries. The course is part of an LMS that allows the administrator to directly assist the users and host forums for deeper discussion. The forum administrators are all members of the M4RD team; technical concerns are managed by Dan Jeffries and content related questions by Lucy Mckay. Where specific expertise for content related questions are required, the team returns to the original authors of Rare Disease 101. Details of these individuals can be found in ‘Additional File 1’. All content can be updated and improved by M4RD to keep it relevant and to reflect feedback. Feedback to date is summarised in the results section of this paper.

## Results

### Rare disease 101

Rare Disease 101 is a unique, interactive e-learning resource aimed at educating clinicians on the management of individuals with rare and undiagnosed diseases. Its approach reflects the notion that rare diseases are individually rare but collectively common; through an introduction to the basics of rare disease as a collective concept, it equips the clinician with an understanding of the impact of living with such diseases and the practical knowledge and tools to be able to manage patients that fall under this umbrella.

**Fig. 1:**
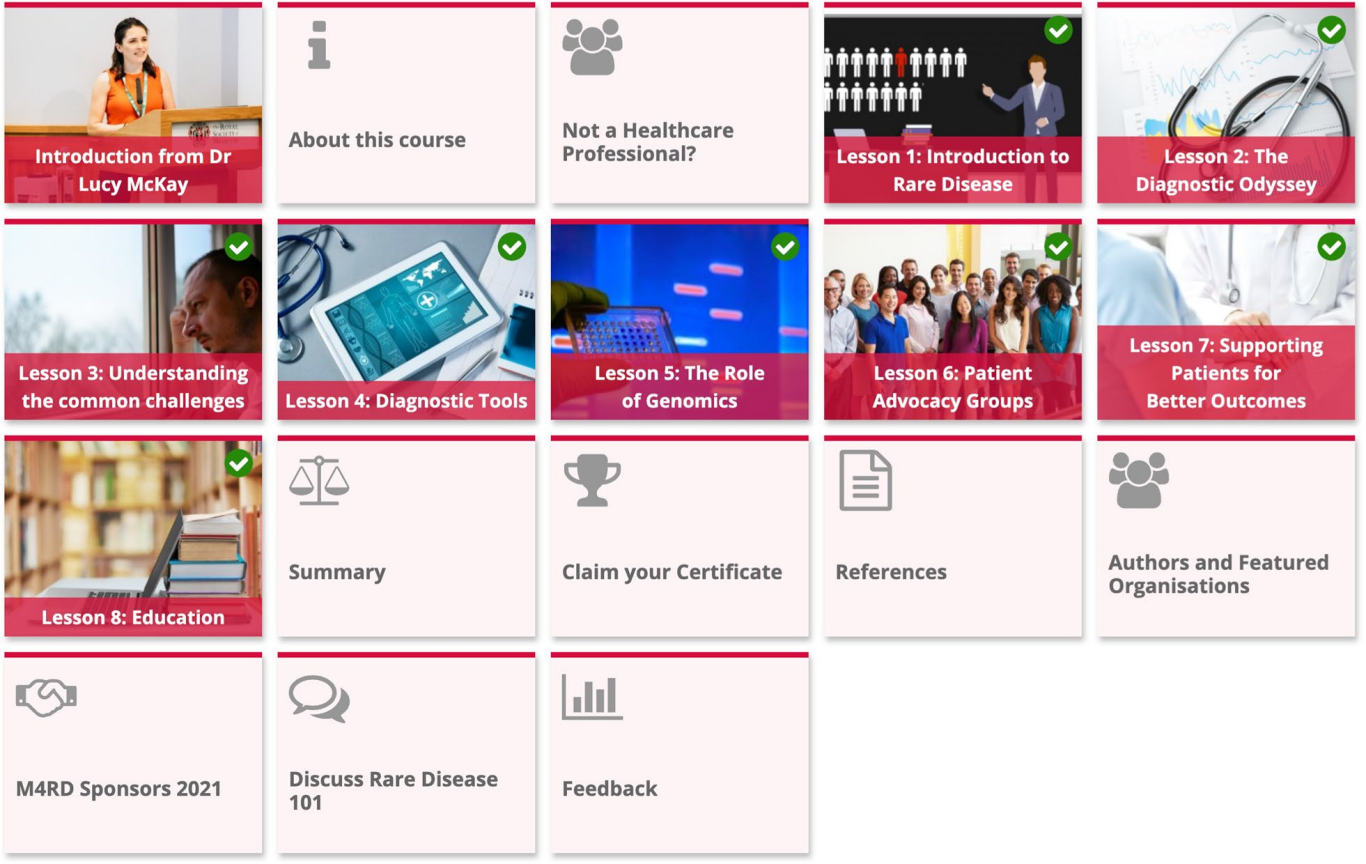
Rare Disease 101 Home page: Overview of content. Available at https://learn.m4rd.org/course/view.php?id=2. Permissions for use by Dr Lucy Mckay, CEO of Medics4RareDiseases

Rare Disease 101 is designed to enrich the knowledge of all clinicians and medical students and thus is freely available to all. On accessing the website the user creates a free account following which Rare Disease 101, and other learning resources, become available. The course is divided into eight individual lessons allowing the learner to complete it over multiple sittings (Fig. 1). Alongside the lessons is an introductory video and information tab to orientate the learner, a summary tab, references, information on sponsors/authors and discussion and feedback tabs. On completion of the lessons a certificate and digital badge are provided, allowing clinicians to utilise this as evidence of additional learning for portfolios.

The eight lessons of Rare Disease 101 lead the learner through their introductory journey into rare disease management in a logical manner (Table 1). Lesson one introduces the concept of rare diseases as a whole, and lesson two the diagnostic odyssey often faced by patients. This is followed by an introduction to the common challenges faced by clinicians and patients within the field of rare diseases (lesson three). Lesson four provides information on pragmatic and useful tools for suspecting when someone has an undiagnosed rare condition and management of rare diseases, improving the clinician’s resources for such consultations. Lesson five explores the role of genomics in rare diseases, highlighting its significant impact on these conditions, and introduces more detailed resources available through Health Education England’s Genomics Education Programme. Lesson six introduces the learner to the vital role of patient advocacy groups in rare disease and lesson seven focuses on how clinicians can support patients for better outcomes from their medical care. The final lesson is on the importance of education in the field of rare diseases, and the role that the learner can play following completion of Rare Disease 101.

To ensure clarity of educational material Rare Disease 101 adopts a simple layout with clear presentation of only key points to prevent information overload. An interactive format is utilised to promote engagement with the material through enhancing visual aids with an element of kinaesthetic learning; lessons are interspersed with short quizzes to allow the learner to assess the knowledge they are acquiring from the course in real time. Patient stories are included to enrich the content of the lessons and to highlight their relevance to clinical practice. To ensure content was accurate and covered a range of perspectives, experts were enlisted from throughout the rare disease community – this included patients, rare disease advocates, clinicians, a geneticist, charities and other organisations.

Ultimately, through a series of short lessons, Rare Disease 101 aims to equip the modern clinician with practical, transferable skills for working with patients in the rapidly growing field of rare disease. The knowledge imparted is specifically tailored to clinical practice, focusing on the basics of rare diseases (content often missed in educating on specific rare diseases) which make up the key concepts for a clinician encountering people living with rare disease in day-to-day practice. The course also introduces the breadth of rare disease medicine through inclusion of, and signposting to, as many patient stories, knowledge sources and tools as possible. Although targeted at doctors/medical students, it is also suitable for allied healthcare professionals.

### Ongoing user feedback

Following completion of Rare Disease 101 users are invited to complete a feedback tab. This information is automatically collated in real time to allow active reviewing of participant opinion of the learning material. This information will allow the team at M4RD to assess the benefit of the material to learners, to review and amend lessons as per feedback and will be used to inform the future directions of Rare Disease 101 including the creation of further lessons.

In the 29 months since Rare Disease 101 went live, 942 individuals have signed up to the LMS and 204 (22%) have completed the full course. The cohort of individuals completing the module was diverse; self-identification of key role demonstrated 76 (37%) participants were medical professionals, 57 (28%) medical students, 24 (12%) other healthcare professionals, 12 (6%) rare disease advocates, 6 (3%) people living with a rare disease, and 29 (14%) other. This showed little variation from the 738 (78%) individuals who have not yet fully completed the course, of which 295 (40%) participants were medical professionals, 126 (17%) medical students, 125 (17%) other healthcare professionals, 66 (9%) rare disease advocates, 30 (4%) people living with a rare disease, and 96 (13%) other.

33 (16%) of the 204 completers have provided formal feedback. The inclusion of this feedback does not account for formal assessment of the tool but provides insight into user opinion to date. Current feedback is limited but positive, with 25 (76%) responses rating Rare Disease 101 ‘excellent’ overall, and the remainder rating it ‘good’. When evaluating applicability of the knowledge attained to the learner’s clinical practice, 18 (55%) stated ‘extremely’, 11 (33%) ‘moderately’ and 2 (6%) ‘somewhat’ (remainder rated ‘not applicable’). All users providing feedback agreed the course is useful in raising awareness of the challenges of living with a rare disease amongst healthcare professionals (19 (58%) ‘extremely useful’, 12 (36%) ‘useful’). A range of aspects were reported as being particularly informative including the importance of active listening in clinical practice, the real-life impact of the diagnostic odyssey, the inclusion of match word tests and knowledge checks, the provision of information on useful tools and patient advocacy groups and one individual reported the genomics lesson provided key information for them to provide answers to some of their patients. Points for improvement were few but included requesting a printable summary handout of key statistics to be included, more information on diagnostic tools and genomics, more information on research in the field and one user mentioning information was repetitive (although ‘great’) – this approach was adopted to ensure the key messages on rare diseases are acquired from the course. In the final feedback section one responder suggested the inclusion of examples of positive and negative clinical encounters, particularly with regards to primary care, to help adoption of good practice and avoidance of bad.

## Discussion

Rare Disease 101 was designed and developed as a means of addressing the gap in formal education in the field of rare disease medicine [5, 8]. The final course consists of eight interactive lessons on aspects of rare disease management, focusing on the common challenges that people living with rare conditions often share [2, 16]. Previous educational tools in academic literature have taught on specific rare diseases and not on the topic as a whole. Rare Disease 101 differs in this aspect; through teaching about the general approach to consulting with a patient with a rare or undiagnosed disease, it aims to equip the clinician with practical tools to succeed in this situation. Lessons are informed by professionals and enriched by patient stories. They introduce important topics of relevance to rare disease medicine such as medical genetics/genomics. External resources and places of support are sign-posted throughout the course in order for Rare Disease 101 to be a jumping-off point to find the expertise required.

Feedback is collected through the same LMS that delivers the Rare Disease 101 lessons. To date, learners have left positive feedback, with all who have rated the material identifying it as either ‘good’ or (mostly) ‘excellent’. They have gone on to say that the knowledge attained through completion of the course is relevant to their practice. This enforces the importance of having formal teaching about the basics of rare disease and identifies Rare Disease 101 as a tool that clinicians feel can help to effectively address this. Feedback evidenced users were from a range of countries and occupations including doctors, nurses, students, patient advocates and (medical) writers.

Rare Disease 101 has a number of strengths. Firstly, it targets a key unmet need, as highlighted by both medical professionals and politicians [2, 5, 18–20]. Secondly, it is a unique tool based in the UK NHS, available through the Department of Health via NHS England’s Genomics Education Programme website. Its international relevance has also been acknowledged; alongside M4RD an Australian government-funded project has seen the rewriting of Rare Disease 101 for the Australian healthcare setting [41], and international organisations such as the UDNI host the learning module online [32]. Thirdly, lessons in Rare Disease 101 are informed by healthcare professionals and patients/patient advocates themselves. This allows the inclusion of information that patients and professionals feel is most important to learners of rare disease management whilst ensuring knowledge is as up-to-date and accurate as possible. The lessons utilise a combination of written, pictorial, video and interactive content, as well as short quizzes, to aid engagement of different learner styles. It assumes no prior knowledge or awareness about rare disease medicine and is clinically-focused, not targeting a specific area of rare disease medicine such as research or drug development [34, 35]. Furthermore, by adopting the e-learning approach utilising an LMS for the course: 1. the tool is easily accessible to a large body of clinicians, globally; 2. content can easily be updated and modified as required; 3. feedback can be collected and collated in real-time, allowing learners to inform the direction of the tool’s further development; 4. a discussion board is run alongside the tool allowing queries and opinions to be shared amongst learners and developers. To date this has included topics such as ‘*Why do rare patients have such a difficult time interfacing with healthcare?*’ and ‘*Suggested actions to raise awareness amongst HCPs—your feedback needed!*’. Completion of the course provides the learner with a certificate to show that they have taken part in further education. This allows additional study to be evidenced in a training portfolio, a common requirement for progression through a medical training pathway. Finally, and most importantly, Rare Disease 101 ensures that all content reflects on the individual affected by the illness, highlighting the importance of patient-centred practice. The use of patient stories as a means of learning promotes the practice of narrative medicine. This transcends the management of patients with rare diseases and trains the learner to better absorb, process and act upon information from the patient journey, ensuring management is tailored to the individual [42]. M4RD will continue to hold live events annually at RSM in order to complement Rare Disease 101 so that users can meet patients and other experts and learn from them in person.

There are a number limitations to Rare Disease 101. Being an online learning resource, it requires access to both a device that can connect to the internet and a good internet connection. This may limit access in some resource-poor settings. Furthermore, the tool itself currently only exists in an English language version meaning learners that do not speak English will struggle to access the tool. Feedback response numbers to date are small, limiting assessment of functionality at this time, and affecting validity of conclusions drawn from survey responses. In addition to this, there is a significant non-completion rate which requires exploration. It is important to note that 63% of those that have not completed the course have yet to commence the first module, indicating many non-completers have yet to begin the course. Completion rate could be improved through marketing/technology by sending reminders to those that have registered, or through introduction of certification for individual modules/groups of modules, not requiring the learner to view all eight topics if they do not wish. Ultimately, a more detailed survey with additional responses would best inform this; this will be explored as M4RD develops its new website which includes a more up-to-date LMS and takes advantage of improvements and greater affordability in this technology since the programme launch. The current version of Rare Disease 101 was a proof of concept proven through RSM; as it has moved online, elements of the course have been integrated into other common resources targeted by professionals for education and training (for example Medscape and NHS England) meaning content will be accessed without formally completing the modules on the M4RD website. Ultimately, the goal of this work is to raise awareness of rare diseases and improve education, regardless of the platform through which the content is accessed. Finally, Rare Disease 101 relies on organic traffic to the site; systematic education will require governing bodies and training institutions to make rare disease education mandatory.

Future steps with Rare Disease 101 include: 1. the addition of further lessons as informed by the requirements of education in rare diseases and feedback from learners; 2. the creation of international versions of the tool to allow non-English speakers to access the modules; 3. highlighting the tool’s relevance to the UK Rare Diseases Framework 2021 as a means of addressing the requirements of clinician education; 4. approaching training institutions to educate teachers on the availability of this resource and the importance of its content to clinical practice.

## Conclusions

Research highlights a lack of disease-agnostic rare disease teaching resources; reports implicate this unmet need in medical education as one contributing factor to the diagnostic odyssey and poor patient outcomes [5, 8, 16]. Rare Disease 101 provides a solution for medical students and doctors in training to have access to a comprehensive, up-to-date educational resource informing on how to manage individuals with rare and undiagnosed diseases. To date this covers the basics of understanding and management of rare diseases; it has the potential for expansion to cover further topics in this area of medical practice. Current feedback of Rare Disease 101 is limited but positive. These early results suggest that the educational programme is well received but further evaluation and assessment is needed. Learners providing feedback felt the resource addressed their academic expectations and spoke positively of the style of the tool, inferring it a suitable model for others to adopt.

### Supplementary Information

Additional file 1.

## Data Availability

Project name: Rare Disease 101. Project home page: https://learn.m4rd.org/course/view.php?id=2. Programming language: HTML5 Package.

## Abbreviations

EJP RD: European Joint Programme on Rare Diseases
EURORDIS: Rare Disease Europe
LMS: Learning Management System
M4RD: Medics 4 Rare Diseases
NHS: National Health Service
NORD: National Organization for Rare Disorders
RSM: Royal Society of Medicine
UDNI: Undiagnosed Diseases Network International
