# Supplementary material for: Rare disease 101: an online resource teaching on over 7000 rare diseases in one short course
Source: Orphanet J Rare Dis. 2024 Jul 22;19:275. doi: 10.1186/s13023-024-03286-8 (PMC11265069; doi:10.1186/s13023-024-03286-8)
Supplement: Supplementary file 1 — Additional file 1. [file 13023_2024_3286_MOESM1_ESM.docx]

**Additional File 1.** Organisations and Individuals involved in content creation for Rare Disease 101

1. **Beacon**; (formerly Findacure) a charity that upskill rare disease patient groups through training, guided programmes, community projects and research initiatives.
2. **Emotive**; creates inspiring programmes that change lives by helping people who are unwell get treatments. 70% of their work focuses on rare diseases.
3. **Rare Revolution**; an independent not-for-profit publisher dedicated to elevating the voice of the rare disease community through its magazine, online presence, disease awareness campaigns, patient engagement projects and social media.
4. **Dr Gemma Chandratillake**; Education and Training Lead for the East of England Genomic Medicine Centre and Director of the Gateway to Genetic Counselling course, part of the Cambridge Genomic Medicine Programme.
5. **Dr Gareth Baynam**; practising Clinical Geneticist, a Clinical Genomics Policy Advisor at WA Health, Director of the Undiagnosed Diseases Program, a co-director of genetic and rare diseases research at the Telethon Kids Institute, and a member of the International Scientific Advisory Board of the new pan-European Union genomic and multi-omic initiative for rare diseases (Solve-RD).
6. **Dr Lucy McKay**; member of a rare disease family, UK trained doctor and rare disease advocate. Founder of the charity Medics4RareDiseases.
7. **Dr Will Evans**; GP with Specialist interest in Clinical Genetics, rare disease advocate, Healthcare researcher and Trustee of Niemann-Pick Uk.
8. **Dan Jeffries**; patient advocate living with two rare conditions. Author and Medics4RareDiseases trustee.
9. **Sarah Lippett**; artist and author. Her autobiographical publications *A Puff of Smoke* (Jonathan Cape, 2019) and *Stan and Nan* (Jonathan Cape, 2016) raise important topics around the health system and family relationships.
